# Health Service Delivery and Economic Evaluation of Limb Lower Bone-Anchored Prostheses: A Summary of the Queensland Artificial Limb Service's Experience

**DOI:** 10.33137/cpoj.v4i2.36210

**Published:** 2021-09-21

**Authors:** D Berg, L Frossard

**Affiliations:** 1 Queensland Artificial Limb Service, Brisbane, Australia.; 2 Your Research Project Pty Ltd, Brisbane, Australia.; 3 Griffith University, Gold Coast, Australia.; 4 University of the Sunshine Coast, Maroochydore, Australia.; 5 Queensland University of Technology, Brisbane, Australia.

**Keywords:** Artificial Limbs, Bionics, Bone-Anchored Prosthesis, Cost-Effectiveness, Cost-Utility, Health Economic Evaluation, Health Service Delivery, Osseointegration

## Abstract

The emergence of skeletal prosthetic attachments leaves governmental organizations facing the challenge of implementing equitable policies that support the provision of bone-anchored prostheses (BAPs). In 2013, the Queensland Artificial Limb Service (QALS) started a five-year research project focusing on health service delivery and economic evaluation of BAPs. This paper reflects on the QALS experience, particularly the lessons learned. QALS' jurisdiction and drivers are presented first, followed by the impact of outcomes, barriers, and facilitators, as well as future developments of this work. The 21 publications produced during this project (e.g., reimbursement policy, role of prosthetists, continuous improvement procedure, quality of life, preliminary cost-utilities) were summarized. Literature on past, current, and upcoming developments of BAP was reviewed to discuss the practical implications of this work. A primary outcome of this project was a policy developed by QALS supporting up to 22 h of labor for the provision of BAP care. The indicative incremental cost-utility ratio for transfemoral and transtibial BAPs was approximately AUD$17,000 and AUD$12,000, respectively, per quality-adjusted life-year compared to socket prostheses. This project was challenged by 17 barriers (e.g., limited resources, inconsistency of care pathways, design of preliminary cost-utility analyses) but eased by 18 facilitators (e.g., action research plan, customized database, use of free repositories). In conclusion, we concluded that lower limb BAP might be an acceptable alternative to socket prostheses from an Australian government prosthetic care perspective. Hopefully, this work will inform promoters of prosthetic innovations committed to making bionic solutions widely accessible to a growing population of individuals suffering from limb loss worldwide.

## INTRODUCTION

My name is Debra Berg. For over 20 years, I have been the manager of Queensland Artificial Limb Service (QALS), a Queensland Health organization delivering artificial limbs to individuals suffering from limb loss. My principal mandate as manager of state service is to support the best possible prosthetic care while ensuring accountability for the use of taxpayer dollars.

### Initial awareness

I became aware that osseointegration could provide opportunities for direct skeletal prosthetic attachment in the early 1990s, when the first cases were presented at various international conferences by Dr. Rickard Branemark, a leading surgeon from the Sahlgrenska University Hospital, Gothenburg, Sweden.^1–3^ Similar to the rest of the prosthetic care community, I recognized the potential capacity of this surgical procedure to alleviate caveats of socket-suspended prostheses (SSPs).^4^ However, it was unclear how contraindications for consumers experiencing vascular problems and the inevitable adverse events (e.g., infections) that could lead to removal of the implant and reamputation should be dealt with.^5^

Regardless, it was clear that the progress of this new treatment was remarkable and truly worth monitoring. Osseointegration was systematically included in QALS' regular horizon scans of prosthetic care innovations having potential to alleviate the clinical and financial burdens of prosthetic attachment for Queenslanders (e.g., review of literature about efficacy and safety).

A handful of patients were first fitted with a screw-type implant in 2000 by a team in Melbourne, Victoria, in collaboration with the pioneering group in Sweden.^6^ Curious to know more, I invited Dr. Kerstin Hagberg, an acclaimed Rehabilitation Specialist from Sahlgrenska University Hospital, to give a talk on her Osseointegrated Prostheses for the Rehabilitation of Amputees (OPRA) study for consumers, clinicians, and healthcare administrators in Brisbane, Queensland, in 2005.^2,7,8^ This presentation gave us a better understanding of the rehabilitation program as well as the benefits (e.g., improvement in health-related quality of life, prosthetic use, embodiment, prosthetic knee and hip range of motion, sitting comfort, donning and doffing, osseoperception, walking ability) and harms of osseointegration (e.g., skin irritation around the stoma, loosening, periprosthetic fractures, mechanical failure of implant parts, deep and superficial infections, removal).^1,7,9^ It also highlighted that bone-anchored prostheses (BAPs) could lessen expenditure from socket fittings and residuum-related skin treatments.^4^ This was the first time I wondered how the emergence of new treatments relying on direct skeletal attachment and the subsequent provision of BAP could impact the day-to-day work of a governmental organization such as QALS.

### Challenges

Answering this question became critical when the first Queenslanders with unilateral transfemoral amputation were treated interstate in late 2012. Initially, we dealt with these consumers on a case-by-case basis. This approach was required to understand and address immediate needs. However, it created too much uncertainty and unpredictability to be sustainable. Furthermore, we anticipated a significant influx of consumers in the short term. Soon after, QALS faced the challenge of putting in place a procedure to warrant a fair and equitable delivery of lower limb BAP to its consumers.

### Needs

As an administrator, and often gatekeeper of taxpayers' money, I considered it essential to make decisions about a new treatment based on the best clinical and socioeconomic evidence available. Prosthetic care must be supported but resources are limited. Like many other managers of government healthcare organizations, every dollar spent by QALS must be spent according to “financial marching orders” (e.g., schedule of allowable expenses).

Literature searches conducted during horizon scans and discussions with colleagues revealed that there was limited information about the alleged socioeconomic advantages of BAP.^10,11^ Clearly, there was a knowledge gap: What could the provision of BAP mean for government healthcare organizations in terms of service delivery and expenditures?

**Figure 1:**
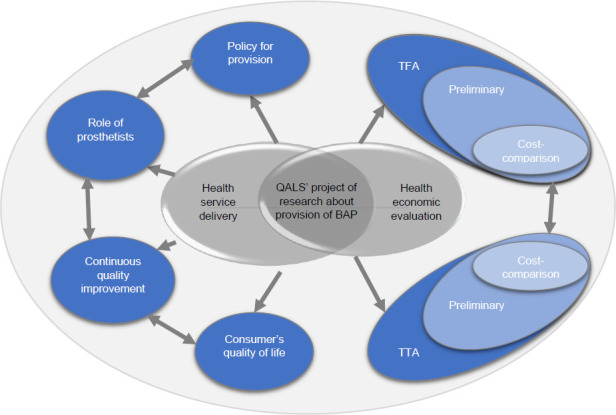
Overview of the research project focusing health service delivery and health economic evaluations of transfemoral (TFA) and transtibial (TTA) bone-anchored prostheses (BAPs) led by the Queensland Artificial Limb Service (QALS).

In 2013, I initiated what turned out to be a five-year project of research gathering evidence to support the provision of BAP from the QALS perspective (**Figure 1**). We assessed the areas of disruptions while trying to find ways to accommodate new expectations.

This project examined changes related to the service delivery of BAP, including the development of a policy supporting the provision of BAP, the role of prosthetists, adjustments of continuous improvement procedures, and consumers' quality of life. This project also involved a health economic evaluation of transfemoral and transtibial BAPs, including cost comparison and preliminary cost-utility analyses (CUAs), compared to SSP.

### Purposes

This paper reflects on the QALS experience gained during this research project. The main purpose was to summarize the outcomes from a bird's-eye view. We have shared the lessons learned during our journey through hands-on information that might be helpful for all BAP promoters, including end users and carers, providers of prosthetic solutions, and administrators of healthcare organizations, amongst others.

The specific objectives were to:

- Introduce some background information about QALS' jurisdiction to facilitate cross-comparison and transferability of our experience
- Outline the drivers that motivated this work
- Present an overview of the impacts and outcomes
- Share the selected barriers and facilitators met during this project separately, although they were intertwined and
- Suggest briefly future developments of this work alongside some calls to action to further promote innovations in the service delivery and economic evaluation of BAP

Drivers, barriers, and facilitators we deemed within and beyond QALS' influence were highlighted so that other organizations could identify their internal strengths and possible external threats during the strategic planning of similar research projects (e.g., strengths, weaknesses, opportunities, and threats analysis).

Supplementary materials to be published in a Data In Brief paper provided additional information about the QALS' jurisdiction, publications (e.g., distribution, breakdown of impacts, downloads worldwide), allowable hours for prosthetist's labor (e.g., phases of treatment, tasks), study cohorts (e.g., sample size, representativeness), and datasets considered to estimate costs (e.g., number of claims, prediction), as well as detailed descriptions of all barriers and facilitators.

## JURISDICTION

QALS is in the jurisdiction of the Queensland State Government Minister of Health, one of the six states and three territories of Australia. The role of QALS is to ensure equitable provision and funding of external prosthetic components to eligible residents of Queensland. Eligible consumers must be registered with the QALS and (1) be eligible for definitive prosthetic funding support under the Queensland Government's “Artificial Limb Scheme” or (2) be eligible under the Rehabilitation Appliance Program of the Department of Veteran Affairs. QALS has a yearly budget of AUD$5.4 million to provide prosthetic services to 3,600 active consumers annually through a network of up to 10 individual prosthetists (e.g., CPO). Although Queensland has predominantly an urban population, QALS services consumers across the whole state.

Queensland has hot and humid weather for the most part of the year. These conditions make the typical SSP difficult to tolerate and increase the need for frequent socket fittings. Access to the closest point of care can be particularly critical for some consumers who might have to travel hundreds of kilometers to visit their prosthetist for socket and component fittings. Altogether, the prospect of socket-free prosthetic solutions could be particularly appealing for QALS consumers.

Currently, QALS is looking after a case-mix of nearly 100 consumers using unilateral, bilateral, and quadrilateral BAPs, representing approximately 11% and 6% of the existing population using BAP which is estimated at 950 in Australia and 1,600 worldwide, respectively. The number of QALS consumers has increased steadily by up to 10 per year over the last three years, generating one of the largest growing populations worldwide.

## DRIVERS

Beyond our initial genuine interest in the economic impacts of the provision of BAP, this research project was pragmatically motivated by a series of external and internal drivers to the organization.

### External drivers

As hinted at in the historical introduction, this project emerged because of external drivers, including, but not limited to, the following:

- A growing number of consumers. In 2012, QALS started to experience a significant influx of existing and new consumers choosing direct skeletal attachments. Projections estimated that the number of consumers choosing BAP will continue to increase noticeably, possibly reaching between 150 and 200 consumers by 2025.
- Prosthetists' concerns. This project was also required to adequately recognize the hours spent by prosthetic care providers looking after consumers with BAP that should be supported by QALS. In 2012, there were no items within the existing QALS' schedules of allowable hours that prosthetists could claim after they provided standard care to fit BAP (e.g., no set hours for a specific service). The pathways for the compensation of their services were unclear. Providers could potentially experience improper compensation for fitting the BAP and loss of revenues from socket fittings.

### Internal drivers

This research project was also needed from several QALS organizational standpoints, including, but not limited to, the need to:

- Apply evidence-based practice. Like other government organizations, QALS was required to provide evidence supporting decisions about reimbursement standards, particularly for the provision of new health technology innovations that could be costly and obsolete within five years.^12^
- Manage stakeholders' expectations. Clarification of the processes for service delivery of BAP was required to manage expectations from QALS stakeholders, including consumers and prosthetic care providers. Consideration whether the proposed procedures had legal bearings might be irrelevant (e.g., unlikelihood of lawsuits). Regardless, QALS believed that outlying these processes should help mitigate potential misunderstandings and conflicts inherent to the implementation of a new and, possibly, risky treatment.
- Verify cost-saving potential. Economic evaluations were required to confirm and, more importantly, to quantify if taxpayers' money could be saved with the provision of BAP, reducing the costs of socket fittings. Understanding cost-saving was essential to facilitate implementation given the budget constraints.
- Assist strategic planning. One of the most critical drivers was to gather sufficient information to complete the QALS' five-year strategic business plan, including yearly budgets for the provision of prosthetic care stratified by case-mix, including those with BAP. It was anticipated that the outcomes of this project would assist QALS with predictable workflow, help manage resources, and ultimately plan a realistic budget.
- Take leadership. Perhaps less pragmatic but equally important was QALS' aspiration to take a leadership role in the area of health economic research on prosthetic osseointegration solutions that was then overlooked.

## IMPACT

### Overview

**Figure 2:**
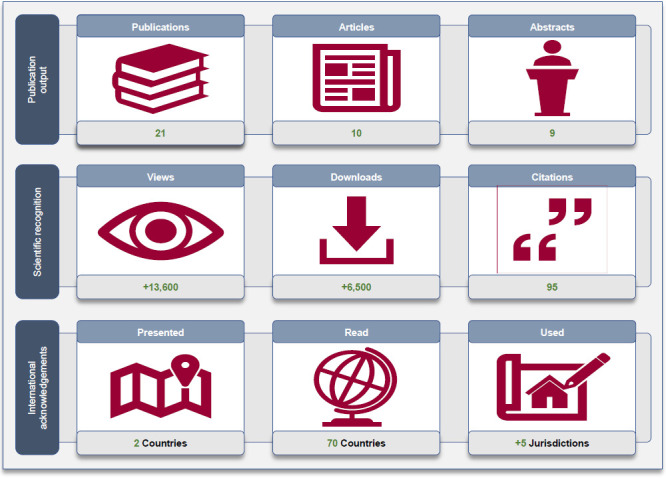
Key indicators of the overall scientific impact of the research project (e.g., publication output, scientific recognition, international acknowledgments) focusing on the health service delivery and health economic evaluation of limb lower bone-anchored prostheses lead by the Queensland Artificial Limb Service between 2015 and 2020.

The overall impact of the project was summarized by nine key indicators which are presented in **Figure 2** reflecting the publication outputs, scientific recognitions, and international acknowledgments.

### Publication outputs

To date, we have authored a series of 21 publications between 2015 and 2020 (e.g., Digital Object Identifier, International Standard Book Number), including six (48%) original research papers, one (5%) dataset paper, three (14%) repository papers, nine (43%) abstracts in national and international conferences, and two (10%) scientific annual reports.^13–33^ Only manuscripts published or in press were considered here. However, several manuscripts are currently in preparation for submission to health economics and prosthetic care journals as well as open access repositories (e.g., Data In Brief).

### Scientific recognitions

The recognition of each publication was assessed using conventional bibliometrics and altmetrics, including the number of views, downloads, and citations extracted from research institutions' repositories, social network sites for scientists, publishers' websites, and citation databases. To date, these publications have accumulated approximately 13,600 views, 6,500 downloads, and 95 citations, as detailed in **Table 1**. Citations of the three papers were in the 46th, 71st, and 46th percentiles corresponding to average, good, and above-average attention scores compared to other papers of a similar age in all journals, according to PharmacoEconomics-Open, Journal of Prosthetics and Orthotics, and Prosthetics and Orthotics International, respectively.^14,16^

**Table 1:** Number and percentage of views, downloads, and citations of each type of publication focusing on the health service delivery and health economic evaluation of limb lower bone-anchored prostheses produced by the Queensland Artificial Limb Service (QALS) between 2015 and 2020.

	Items		Views^(1)^		Download^(1)^		Citations^(1)^	
	(#)	(%)	(#)	(%)	(#)	(%)	(#)	(%)
**Total publications**	**21**	**100**	**13,666**	**100**	**6,543**	**100**	**95**	**100**
**Total papers**	**10**	**48**	**9,859**	**72**	**5,298**	**81**	**94**	**99**
Original papers ^(2)^	6	29	9,638	71	5,144	79	91	96
Dataset papers ^(3)^	1	5	221	2	154	2	3	3
Repository papers ^(4)^	3	14	0	0	0	0	0	0
**Total abstracts**	**9**	**43**	**2,930**	**21**	**841**	**13**	**0**	**0**
International conference ^(5)^	1	5	243	2	62	1	0	0
National conference ^(6)^	8	38	2,687	20	779	12	0	0
**Total reports**	**2**	**10**	**877**	**6**	**404**	**6**	**1**	**1**

^(1)^ Extracted from research institutions' free-access repositories (i.e., Queensland University of Technology's ePrint, University of the Sunshine Coast's Research Banks, Griffith University Research Online), social networks sites for scientists (i.e., ResearchGate, Mendeley), publishers' websites (i.e., Canadian Prosthetics & Orthotics Journal, Data In Brief, Journal of Prosthetics and Orthotics, PharmacoEconomics-Open, Prosthetics and Orthotics International) and citation databases (i.e., Google Scholar, Elsevier's Scopus); ^(2)^ Published in Canadian Prosthetics & Orthotics Journal, Journal of Prosthetics and Orthotics, PharmacoEconomics-Open, Prosthetics and Orthotics International, The AOPA Revie; ^(3)^ Published in Data In Brief; ^(4)^ Published in Mendeley; ^(5)^ Presented at International Society of Prosthetics and Orthotics; ^(6)^ Presented at Australasian Osseointegrated for Amputees Conference

### International acknowledgments

Analyses of ePrint records indicated that publications were downloaded from approximately 70 countries, with 75% of the downloads made from Australia (32%), United States of America (30%), Canada (6%), United Kingdom of Great Britain and Northern Ireland (4%), and Ireland (3%). More importantly, these publications were considered and often cited in recent health technology assessments of osseointegrated prosthetic solutions produced by several government organizations (e.g., Australian states, Canadian provinces, United Kingdom, New Zealand, Spain).^34–40^ This work provided guidance when the Australian National Disability Insurance Scheme developed its funding model.

## CONTRIBUTIONS

The actual developments of each topic of research progressed altogether and often organically, depending on opportunities and resources. Therefore, contributions are presented by topics rather than historical evolution.

### Health service delivery

Our primary contribution was the development of a policy regulating the provision of BAP-specific prosthetic care. Effectively, these procedures organized a workflow meshing role for prosthetists, a quality improvement of specific procedures, and assessment of overall consumers' experience and quality of life.

### Policy for provision of BAP

In 2012, information from health technology assessments of direct skeletal attachment that could help develop this policy was sparse.^10,11,41–44^ Consequently, we conducted an action research study involving the first 18 QALS consumers between January 2011 and June 2015 to create QALS policy for the provision of transfemoral BAP.^14,23,28^

An initial version of this policy was published in 2017 (e.g., tasks, documents, costs), including possible obstacles and facilitators to implementation.^14^ An equitable provision of transfemoral BAP was based on seven processes involving fixed expenses during the treatment and five processes regulating ongoing prosthetic care expenses. The cornerstone of this policy was the allowance of 22 h toward prosthetist's labor to support delivery of BAP care costing up to AUD$3,300 per consumer. A prosthetist could spend 2.5 h (11%), 2.5 h (11%), 6.5 h (30%), and 10.5 h (48%) during the preoperative, surgical, fitting of light and definitive limb prostheses, and postoperative phases of the treatment, respectively.

This policy required adjustments related to the prosthetists' scope of practice, funding of prosthetic limbs during rehabilitation, and allocation of microprocessor-controlled prosthetic knees.

### Role of prosthetists

Early investigations revealed that the role of prosthetists in the provision of BAP has been largely overlooked, although they are at the heart of treatment (e.g., primary point of contact for consumers, responsible for prosthetic loading).^10,17,42^ In the policy presented earlier, prosthetists could claim up to 22 h of labor including 4 h (18%), 2 h (9%), 14 h (64%), and 2 h (9%) to consult with the clinical team, evaluate functional outcomes, fit light and definitive prostheses, and report progress to stakeholders before and after the surgical implantation of the osseointegrated fixation, respectively.^14^

**Figure 3:**
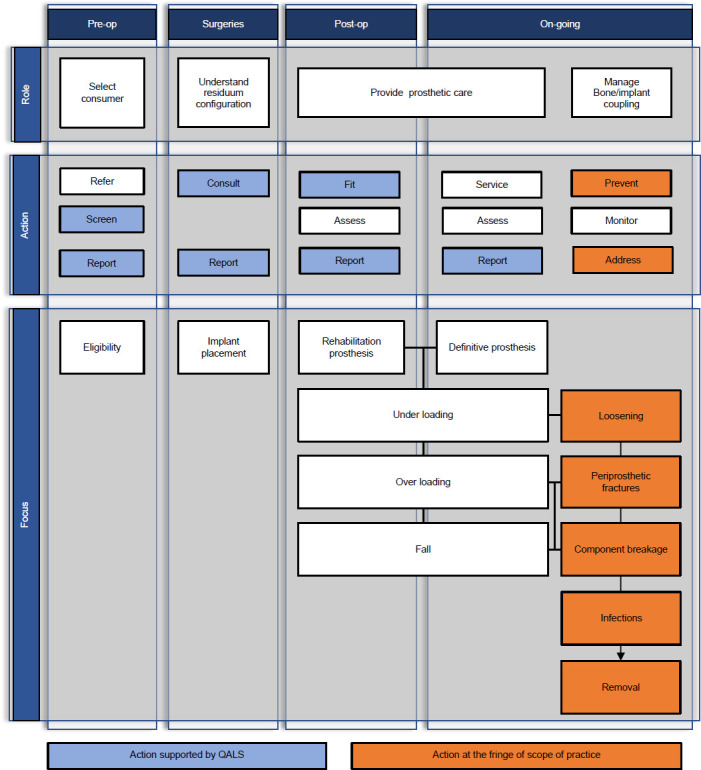
Roles, actions and focus of prosthetic care provided by prosthetists to consumers fitted with transfemoral and transtibial bone-anchored prostheses at various stages of treatment (e.g., pre-op, surgery, post-op, on-going) included tasks supported by Queensland Artificial Limb Service (QALS) and tasks of the fringe of usual scope of practice of prosthetists. Adapted from Frossard et al (2018).^17^

As summarized in **Figure 3**, Frossard et al. (2018) further detailed the critical roles prosthetists could play during the provision of BAP, including referral of consumers (e.g., discussing fitting options, elucidating surgical procedures, selecting the surgical team).^17^ The survey presented by Frossard et al. (2019) indicated that 25% of QALS consumers found information about the surgical procedure from a prosthetist.^20^ As expected, prosthetists should be responsible for usual fitting tasks (e.g., selection of components, alignment of prosthesis, prevention of falls). However, as reported in Clark (2021), prosthetists also play a key role in the prevention of load-related adverse events when fitting bone-anchored bionics prostheses.^45^ Fittings of BAP must be made with additional constraints to limit unwanted loads, leading to increased risks for the bone-implant interface (e.g., loosening, breakage of connector and safety device, periprosthetic fractures, infection, removal).^46–50^

Altogether, this study showed that the provision of BAP has the potential to be slightly outside the usual scope of practice of prosthetists.^51^ Training opportunities by qualified experts, guidelines from suppliers of implants, and formal recommendations from governing bodies about prosthetic care of consumers fitted with BAP and business management that could help reduce risks are sparse, or even missing, in some jurisdictions. Prosthetists may potentially be exposed to increased risks when treating BAP consumers.^17^

### Quality improvement procedure

The implementation of the QALS policy for the provision of transfemoral BAP has subsequently created a need for a continuous quality improvement (CQI) procedure seeking to enhance consumers' experience with the QALS process, supporting the provision of BAP.

**Figure 4:**
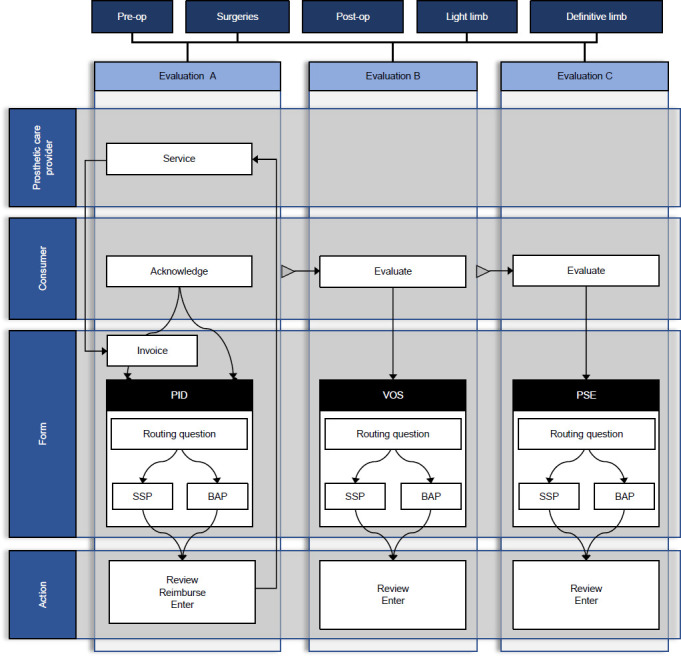
Overview of continuous quality improvement (CQI) procedure seeking to enhance Queensland Artificial Limb Service (QALS) consumer' experience with the provision of socket-suspended (SSP) and bone-anchored (BAP) prostheses that involved collection of data with prosthetic care providers and consumers (PID: Prosthetic Issue Document, VOS: Validation of Services, PSE: Prosthetic Service Evaluation). Adapted from Frossard et al (2018).^17^

Frossard et al. (2018) presented a BAP-inclusive CQI procedure.^17,32^ A redesign study led to this procedure to collect, analyze, and report the experience of 65 QALS consumers who delivered BAP-specific prosthetic care, as presented in **Figure 4**. The proposed CQI procedure required 1.3 h of prosthetist labor or 6% of the 22 h allowed for the whole procedure presented above, costing AUD$213 per episode of care. The time spent by a prosthetist, consumer, and QALS staff represented 24%, 24%, and 53% of the CQI procedure, respectively. The costs of labor for prosthetist and QALS staff represented 70% and 30% of the CQI procedure, respectively.

This study demonstrated that government organizations can redesign a CQI procedure for comprehensive appraisal of the provision of prostheses that could be: inclusive of BAP, affordable and swift for prosthetists. Achieving a minimally disruptive BAP-inclusive CQI procedure can be facilitated by adaptation of a procedure already in place (e.g., use of routing questions to indicate if the survey is for SSP or BAP).

### Consumer's quality of life

Another integral part of the CQI procedure was to assess consumers' experience with the overall provision of BAP and changes in their quality of life after implantation of an osseointegrated fixation.

Frossard et al. (2019) presented the outcomes of a 25-question ad hoc survey, including 7 (28%), 5 (20%), and 13 (52%) questions about “Osseointegration Surgery Details,” “Pre-Osseointegration Surgery,” and “Post-Surgery Osseointegration”, respectively.^20^ A total of 12 out of the 65 eligible QALS consumers completed the survey, giving a return rate of 18%. All respondents were “happy” with their BAP and indicated that “it works as it should”, including 91% of respondents satisfied with the componentry fitted to their BAP. Key figures of the respondents' experience with efficacy and safety of the procedure are provided in **Table 2**. More importantly, all respondents reported a level of satisfaction and quality of life above eight and seven out of 10 after surgical implantation of the osseointegrated fixation and fitting with BAP, respectively. These outcomes suggest that QALS policy about the provision of BAP seemed to contribute favorably to overall consumer satisfaction.

**Table 2:** Key figures about the efficacy and safety of surgical implantation of the osseointegrated fixation and fitting with boneanchored prosthesis (BAP) extracted from self-reported ad hoc consumers survey administered by Queensland Artificial Limb Service.

Efficacy	Safety
Respondents wear their BAP on average 17±6 hours per day 91% of respondents said their BAP supported their lifestyle needs	58% of respondents experienced some infections around the exit point of their percutaneous part post-surgery Respondents experience an episode of infections the exit point of their percutaneous part post-surgery for an average of

Altogether, this work provided benchmark information that can educate the design of patients' experience surveys and clinical trials looking at the effects of bionic solutions on consumers' quality of life (e.g., built-in governmental CQI procedure).

### Health economic evaluations

The QALS policy was validated by economic evaluations. Basically, this involved looking at CUA comparing BAP (new interventions) and SSP (usual treatment) using the incremental cost-utility ratio (ICUR) based on incremental costs, expressed in Australian dollars, and utilities, expressed in quality-adjusted life-years (QALYs), over time, that could be compared to the willingness-to-pay threshold (WTP) set at AUD$40,000 per QALY.^16,18,52–55^

**Figure 5:**
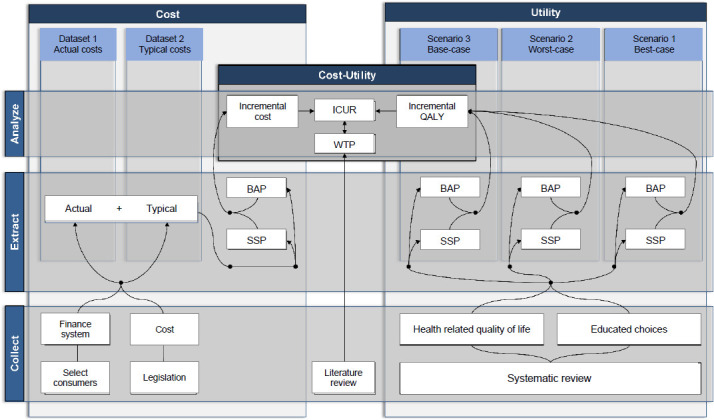
Overview of the approach applied to conduct preliminary cost-utility analyses (CUA) of transfemoral and transtibial bone-anchored (BAP) compared to socket-suspended (SSP) prostheses providing incremental cost-utility ratios (ICUR) based on incremental costs expressed in monetary units and utilities expressed in quality-adjusted life-years (QALY) that were compared to willingness-to-pay threshold (WTP) for small series of plausible scenarios (e.g., base-case, worst-case, best-case) over a six-year time horizon from Queensland Artificial Limb Service (QALS) prosthetic care perspective.^16,18^

We purposely chose to perform preliminary CUAs, as detailed below, when discussing barriers and facilitators. These analyses were conducted following an initial version of the 15-step iterative process (e.g., feasibility, constructs, analysis, interpretation) presented by Frossard et al. (2021).^38,39^ Both preliminary CUAs of transfemoral and transtibial BAPs were performed for a small series of plausible scenarios over a six-year time horizon from the government perspective.^16,18^ An overview of our approach to collect, extract, and analyze estimates of costs and utilities is presented in **Figure 5**. Total costs combined actual and typical costs extracted from financial records and allowable expense schedules, respectively. Baseline utilities were extracted from the literature, while incremental utilities were assumed.

### Preliminary cost-utility analysis of transfemoral BAP

Frossard et al. (2017) cross-compared historical costs for the provision of SSP with the simulated costs for transfemoral BAP (e.g., labor, parts).^14^ Costs were extracted from QALS regulatory documentation according to functional levels (e.g., K-levels) and low-cost, budget, and high-cost options for knee and ankle units. The provision of a transfemoral BAP was 18% and 79% less than SSP for the prosthetist labor and attachment costs, respectively. BAP was more economical by AUD$18,200, AUD$7,000, and AUD$1,600 when fitted with low-cost, budget, and high-cost options, respectively, compared with SSP for the highest functional level (i.e., K4).

Frossard et al. (2018) reported preliminary CUA for a cohort of 16 QALS consumers using transfemoral BAP (**Table 3**).^16^

**Table 3:** Outcome of preliminary cost-utility analyses providing indicative incremental cost-utility ratio (ICUR) based on incremental costs expressed in Australian dollars and utilities expressed in quality-adjusted life-years (QALY) for the provision of transfemoral (2016-2017 prices: 1 Australian dollar ≈ 0.71 Euro ≈ 0.60 British pound ≈ 0.76 US dollar) and transtibial (2018-2019 prices: 1 Australian dollar ≈ 0.63 Euro ≈ 0.54 British pound ≈ 0.71 US dollar) bone-anchored prosthesis (BAP) from Queensland Artificial Limb Service (QALS) prosthetic care perspective (N: Number of consumers).^16,18^

	Increment cost ($/cycle)	Increment utility	Indicative ICUR ($/QALY)
**Transfemoral BAP (N=16)**			
Mean	$13,562	0.815	$16,632
Standard deviation	$16,497	0.000	$20,231
Minimum	−$20,933	0.815	−$25,671
Maximum	$43,625	0.815	$53,499
**Transtibial BAP (N=12)**			
Mean	$5,604	0.489	$11,453
Standard deviation	$12,180	0.000	$24,895
Minimum	−$12,263	0.489	−$25,065
Maximum	$20,514	0.489	$41,929

**Figure 6:**
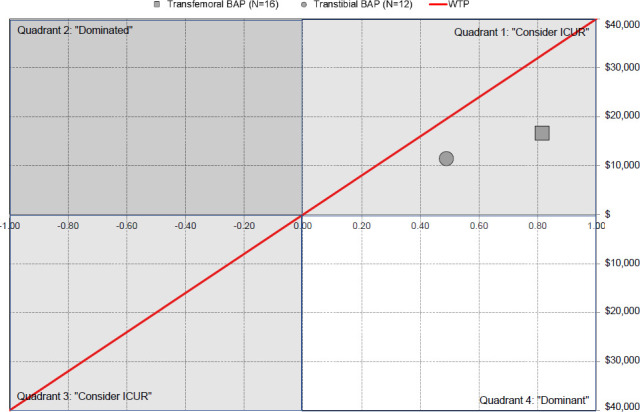
Overview of cost-utility analysis showing indicative incremental cost-utility ratio of AUD$16,632 and AUD$11,453 per quality-adjusted life-year (QALY) and willingness-to-pay threshold (WTP) of AUD$40,000 per QALY for transfemoral (2016-2017 prices: 1 Australian dollar ≈ 0.71 Euro ≈ 0.60 British pound ≈ 0.76 US dollar) and transtibial (2018-2019 prices: 1 Australian dollar ≈ 0.63 Euro ≈ 0.54 British pound ≈ 0.71 US dollar) bone-anchored prosthesis (BAP) that were more costly and more effective than socket-suspended prosthesis (Quadrant 1) and below WTP from Queensland Artificial Limb Service's (QALS) prosthetic care perspective, respectively (N: Number of consumers)**.^16^,^18^**

The average cost for the provision of transfemoral BAPs was approximately 40% (AUD$13,562±AUD$16,497) more than SSP, which can be partially offset by an increase of 0.815 QALY. The provision of a transfemoral BAP was cost-saving and cost-effective for 19% and 88% of the consumers, respectively. The indicative ICUR for the provision of a transfemoral BAP was approximately AUD$17,000 per QALY and significantly below the WTP (**Figure 6**).

### Preliminary cost-utility analysis of transtibial BAP

Frossard et al. (2021) reported a preliminary CUA for six QALS consumers using transtibial BAP (**Table 3**).^18^ The average cost for the provision of transtibial BAPs was approximately 20% (AUD$5,604 ± AUD$12,180) more costly than SSP, which can be offset by an increase of 0.489 QALY. The provision of a transtibial BAP was more expensive and cost-saving for 67% and 33% of the participants, respectively.

The indicative ICUR for the provision of a transtibial BAP was AUD$12,000 per QALY and significantly below the WTP (**Figure 6**).

### Early evidence of health economic benefits

These studies revealed that early engagements with suppliers of prosthetic components particularly suited for transfemoral BAP can strongly impact the overall costs (e.g., economical advanced knee and foot/ankle units). These studies also highlighted that suppliers of osseointegrated fixations can influence the outcomes of CUA as the cost of their percutaneous parts (e.g., connectors, protective device) could offset the costs of socket fittings. In all cases, the provision of both transfemoral and transtibial BAPs appeared to be acceptable alternatives to SSP from an Australian government prosthetic care perspective.

## BARRIERS

An overview of the 17 main barriers encountered during this project is presented in **Table 4**. A total of 5 barriers (29%) were related to service delivery, 11 (65%) to economic evaluation, and 5 (29%) to project management. A total of 4 and 13 barriers were deemed unlikely (e.g., access to limited resources, dealing with multiple funding allowances, addressing ethics issues, accommodating new national schemes) and likely (e.g., face inconsistency of care pathways, design preliminary CUA, predict timeline of publications) to be met by other government organizations such as QALS. Here, we have only detailed the core barriers that set in motion cause-and-effect reactions onto other obstacles.

**Table 4:** List of common and QALS barriers related to health service delivery (HSD) and/or health economic evaluation (HEE) and/or project management (PM) encountered during research focusing on the provision of lower limb bone-anchored prostheses (BAP) led by the Queensland Artificial Limb Service (QALS). ((S): Detailed description to be published in a Data In Brief).

		HSD	HEE	PM
**1**	**QALS barriers**			
1-1	Access to limited resources			x
1-2	Deal with multiple funding allowance ^(S)^			x
1-3	Address ethics issues ^(S)^	x	x	
1-4	New national scheme ^(S)^			x
	**Number of QALS barriers**	**1**	**1**	**3**
**2**	**Common barriers**			
2-1	Face inconsistency of care pathways	x	x	
2-2	Sort out schedules of allowable expenses	x	x	x
2-3	Deal with diversity of outcome measures	x		
2-4	Palliate limited standards of prosthetic care	x		
2-5	Choose health economic analysis ^(S)^		x	
2-6	Choose type of cost-utility analysis ^(S)^		x	
2-7	Design preliminary cost-utility analysis		x	
2-7-1	Choose relevant perspective		x	
2-7-2	Establish relevant time horizon		x	
2-7-3	Estimate costs		x	
2-7-4	Access utilities		x	
2-7-5	Estimate weight of assumptions		x	
2-8	Predict timeline of publications ^(S)^			x
	**Number of common barriers**	**4**	**10**	**2**
	**Number of barriers**	**5**	**11**	**5**

### Access to limited resources

As with most prosthetic care departments, resources to undertake a research project of developing evidence-based policy are sparse. Unfortunately, we were unable to collaborate with services specialized in health technology assessment within the Minister of Health. In 2016, we applied for two unsuccessful grants (e.g., Defense Health Foundation Grants for Medical Research, Australian Centre for Health Services Innovation – Implementation Grant). Supports from other services and funders were curtailed by their perception that the provision of BAP was “too niche.” Alternatively, the project was to run with QALS and its partner resources (e.g., staff time, consultancy).

### Face inconsistency of care pathways

Another root cause barrier was the unpredictability of BAP care pathways corresponding to the onset of a series of interventions made by specialists during the course of treatment. Generic descriptions of the surgical procedures and rehabilitation programs specific to either screw-type or press-fit implants published by teams overseas were available when we started.^1,2,,6,7,9,56–62^

**Figure 7:**
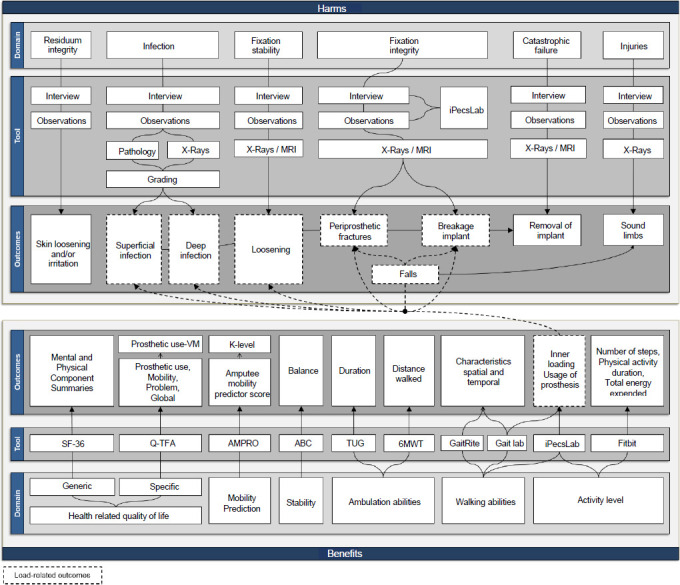
Overview of evaluation framework to extract clinical benefits and harms including prosthetic load-related outcomes (SF-36: 36-Item Short Form Survey, Q-TFA: Questionnaire for Persons with a Transfemoral Amputation, AMPRO: Amputee mobility predictor, ABC: Activities-specific Balance Confidence scale, TUG: Timed Up and Go test, 6MWT: 6-minute walk test, GaitRite (CIR Systems Inc, USA), Gait laboratory equipment (e.g., 3D motion capture, force plates), iPecsLab (RTC electronics, USA), Fitbit (Fitbit Inc, USA)).^17,63^

Additional ad hoc guidance for specific aspects were provided regularly by main teams in Australia as their own procedure evolved organically from case to case. Sometimes information from various sources agreed, but they often contradicted themselves. Consumers in the same case-mix rarely followed comparable care plans. Practically, it was difficult to grasp “who was doing what and when” around the fitting of BAP. Uncertainty about the continuum of care across preoperative, surgical, and postoperative phases of the treatment created the following barriers:

- Sort out schedules for allowable expenses. The adequate allocation of allowable hours to support the provision of BAP-specific prosthetic care was initially complicated by the lack of clarity of overall care pathways. Furthermore, providers expressed legitimate concerns about the economic viability of delivering BAP, reducing revenue from socket fittings. Discussions with clinical teams and prosthetists led to a consensus and subsequent creation of the QALS schedule, including 22 allowable h of labor to support BAP care. This was only approximately 10 h less compared to the typical 32 h of labor allowed for a socket fitting (i.e., 6 h to cast the residuum, 20 h to build a socket, 6 h to fit a socket).^14,17,38,39^ However, the loss of income could be compensated by fitting BAP with high-end components.
- Dealing with diversity of outcome measures. Accessing clinical outcomes with osseointegrated implants is critical for health economic evaluations (e.g., choice of utility). However, assessing benefits, let alone harms, of surgical treatment was beyond QALS' prerogatives. Alternatively, we had to rely on a limited number of outcomes extracted from external sources. Choosing relevant outcomes was facilitated by the generic evaluation framework presented in **Figure 7** which mapped out standardized and nonstandardized instruments for quantitative or qualitative measures of the benefits and harms before and after the fitting of BAP used by teams overseas.^17,63^ Ultimately, we preferred health-related quality of life data measured by the standardized 36-Item Short Form Survey (SF36) as the primary outcomes to reflect benefits and, more particularly, utility of the treatment.^7,57,64^
- Palliate limited standards of prosthetic care. Inconsistent care pathways and diversity in outcome measures, all combined, hinder the understanding of the cause-and-effect relationships between treatment options, benefits, and harms (e.g., two-stage for screw-type, single-stage for press-fit).^65,66^ This limited the emergence of reasonable standards for BAP-specific prosthetic care, let alone the best standards around fitting arrangements that could possibly maximize benefits and minimize exposure to risks (**Figure 7**). However, the evaluation framework raised our awareness about the links between the risks of adverse events and loading regimen depending on the fitting of components as well as daily usage of BAP. Clearly, the choice of components can play a critical role in reducing load-related harms susceptible to osseointegration and the long-term stability of the bone/implant coupling.^46,48–50,67–69^ Initially, only a small case series showed differences between loading profiles applied by different categories of components (e.g., basic and advanced knee units).^69^ We examined mechanically passive components with basic functions such as single-axis or polycentric hydraulic knees and multiaxial foot-ankle units. Finally, we acknowledged that the fitting of the microprocessor-controlled knee (MPK) and energy-storing-and-return (ESAR) foot was required. This decision was based on the best evidence available and, more heavily, on the alleged capabilities of these components to increase stability (e.g., stance and swing control), ease of walking (e.g., high range of motion, mechanically powered push-off), attenuate excessive loading (e.g., auto-adaptive stance and swing phases), and reduce falls (e.g., automatic stumble recovery).^70^ Ultimately, we opted to support the provision of a “budget option.” This package combines a single-axis cadence-responsive knee, shock absorption adapter, tube adapter, and a dynamic foot that are commonly provided to QALS consumers with the highest functional outcomes (e.g., K4).

### Design preliminary CUA

Undertaking preliminary CUA came with a range of subsequent obstacles to overcome when choosing the constructs framing the analysis, including, but not limited to, the following:

- Choosing a relevant perspective. First, we had to choose the perspective of the CUA corresponding to the point of view adopted when deciding which healthcare costs should be considered. In principle, a comprehensive analysis could include all surgical, medical, and prosthetic healthcare costs covered by taxpayers. However, Queensland State healthcare organizations are structured in such a way that whether the fitting of BAP affects medical costs has little impact on the QALS' resources. We were more concerned with the potential reduction in the cost of prosthetic care. Therefore, CUAs were only conducted from the perspective of government prosthetic care.
- Establishing a relevant time horizon. The second obstacle was to determine the relevant time horizon corresponding to the time over which outcomes of the innovation should be evaluated.^71,72^ Basu et al. (2019) stated that the time horizon must be long enough to capture the intended and unintended benefits and harms of the intervention.^71^ O'Mahony et al. (2015) indicated that it is often unclear how time influences both the technical adequacy of cost-effectiveness analyses and their correspondence to the policy choices they seek to inform.^73^ Osseointegrated implants are permanent, and fittings of BAP are continuous. At first glance, it could make sense to perform a comprehensive CUA using Markov decision-analytic models to look at multiple scenarios over scalable time horizons (e.g., years, decades, lifetime).^44,74–78^ However, O'Mahony et al. (2015) demonstrated that the approximation error is larger with the long cycle length and that the short cycle cost-effectiveness analyses better approximates the continuous-time reality.^73^ Furthermore, the World Health Organization recommended the production of generic cost-effectiveness analyses focusing on resources that could realistically be reallocated over the time horizon of the analysis.^72^ These recommendations lead us to make a compromise of a six-year time horizon, allowing a reasonable prediction of the costs over the components' life cycle (e.g., two cycles of three years for a foot, three cycles of two years for a knee).^16,18^
- Estimate costs. Expenses from the QALS financial system for the provision of SSP or BAP were unavailable when individuals became QALS consumers less than six years before the surgery or when surgery occurred less than six years before the end of the study. As detailed above, the total costs were estimated by blending actual and typical costs. A prediction variable corresponding to relative typical costs over the total costs, expressed as a percentage of the six-year funding cycle, was created to specify the level of uncertainty of the cost estimates. A prediction of 0% and 100% indicated that the total costs were fully extracted from the schedule and financial records, respectively. The overall cost predictions were 48±20% and 46±22% for the provision of transfemoral (SSP, 42±32%; BAP, 55±27%) and transtibial (SSP, 43±40%; BAP, 49±12%) prostheses, respectively.
- Access utilities. In principle, utility data may have been obtained from the Australian treating teams. However, this option turned out to be impractical (e.g., access limited by ethics, no state-based stratification of datasets) and potentially unreliable (e.g., no clinical trial registration). These issues were resolvable. However, we chose to consider the quality of life status published previously.^7,8^ Baseline QALY were extracted from SF36 datasets converted into QALY applying regression model.[16, 18] We made conservative assumptions to determine the incremental gain of QALY between the SSP and BAP fitting options.
- Estimate the weight of the assumptions. By definition, preliminary CUAs overlook comprehensive uncertainty and sensibility analyses. Therefore, understanding the impact of assumptions to estimate individual costs (e.g., creation of a schedule of allowable expenses, blending of actual and typical costs) and utilities (e.g., extraction of baseline from literature, assumptions for incremental gain) on both ICURs for transfemoral and transtibial BAPs was limited.

The choice of the preliminary CUA turned into a facilitator over time. Shortcomings might limit the strength of the evidence of cost-utility. However, this decision was critical in delivering the project on budget, on time, and with added value. Furthermore, publications of the outcomes contributed to the conversation about the relevance and possibly the standardization of preliminary CUAs to assess prosthetic care innovations.^38,39,79–81^

## FACILITATORS

An overview of the 18 key facilitators is presented in **Table 5**. A total of 4 (22%) facilitators related to service delivery, 10 (56%) to economic evaluation, and 8 (44%) to project management.

**Table 5:** List of specific and transferable facilitators related to health service delivery (HSD) and/or health economic evaluations (HEE) and/or project management (PM) encountered during research focusing on the provision of lower limb bone-anchored prostheses (BAP) led by the Queensland Artificial Limb Service (QALS). ((S): Detailed description to be published in a Data In Brief)

		HSD	HEE	PM
**1**	**QALS facilitators**			
1-1	Engage with local research teams ^(S)^			x
1-2	Involve critical number of consumers ^(S)^	x	x	
1-3	Access to financial data ^(S)^		x	
1-4	Customize database		x	
1-4-1	Import historical data		x	
1-4-2	Code expenses		x	
1-4-3	Compare costs		x	
1-4-4	Create reports		x	
1-5	Share datasets ^(S)^			x
1-6	Use of free repositories			x
	**Number of QALS facilitators**	**1**	**7**	**3**
**2**	**Transferable facilitators**			
2-1	Frame action-research plan			x
2-1-1	Gather reference group	x	x	x
2-1-2	Create stakeholder matrix			x
2-1-3	Profile case-mix	x	x	
2-2	Adapt rather create procedure ^(S)^	x		
2-3	Choose preliminary CUA ^(S)^		x	
2-4	Engage with social media ^(S)^			x
2-5	Monitor impact ^(S)^			x
	**Number of transferable facilitators**	**3**	**3**	**5**
	**Number of facilitators**	**4**	**10**	**8**

A total of 10 facilitators might be specific to QALS (e.g., engage with local research teams, involve a critical number of consumers, access to financial data, customize databases, share datasets, use of free repositories). Eight facilitators could be transferable to other organizations (e.g., frame action research plan, choose preliminary CUA, adapt rather than create procedures, engage with social media, monitor impact). Next, we only detailed the facilitators deemed the most critical.

### Customize database

QALS' preliminary CUAs were facilitated by a piece of software purposely designed to:

- Import historical data from 1,840 vouchers exported from QALS' financial system for CUA of transfemoral (i.e., 1,598 vouchers) and transtibial (i.e., 242 vouchers) BAPs.^16,18^
- Code individual expenses from 4,014 claims to identify whether there were for transfemoral or transtibial prostheses, SPP or BAP, labor (e.g., fitting prosthesis) or parts (e.g., prosthetic knees and feet units), attachment (e.g., socket, connectors), or prosthesis.^16,18^
- Compare aggregated costs for individuals and groups over the time horizon with SSP and BAP before and after surgical intervention, respectively.^14,16,18^
- Create reports including tables and figures formatted for internal communication (e.g., quarterly budget, annual reports) and publications of papers (e.g., manuscript, supplement).^14,16,18^

This database gave us the flexibility to run queries on demand to present the most up-to-date analyses and outcomes (e.g., new individual expenses to improve predictions).

### Use of free repositories

Like most government organizations, QALS must make the outputs of the project freely available to taxpayers in Australia and elsewhere, in a timely manner.

We made the point to share original research, datasets, and repository papers including supplements and spreadsheets as well as abstracts and scientific annual reports available free of cost to the public, either from publishers' websites, social network sites for scientists, and/or research institution repositories (**Table 1**).

Availability of publications increased visibility and built up credentials. Portals provided a means to monitor the impact (e.g., numbers of views and downloads). In the long run, we hope that access to primary information will encourage collaboration with other promoters of BAP and facilitate secondary observational studies (e.g., analyses of cause-effect relationships between confounders and provision of BAP) and literature reviews and meta-analyses.40

### Frame action research plan

**Figure 8:**
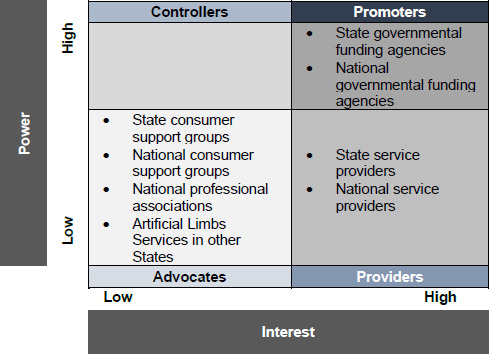
Typical stakeholder matrix including groups of controllers, promoters, providers and advocates of the procedure depending on power (e.g., capacity to influence allocation of resources) and interest (e.g., capacity to provide prosthetic and medical care). Adapted from Frossard et al (2018).^17^

Perhaps more transferable were the lessons learned from the first steps of action research. Studies started with the planning phase, including practical tasks to define the project (e.g., identify problems to solve, root cause analysis, define objectives, profile case-mix), determine the deliverables (e.g., review regulatory obligations, conduct stakeholder's analysis, determine reporting expectations), and review the literature. The following tasks were particularly helpful and transferable:

- Gathering a reference group or a “think tank” including experts in service delivery, health economics, data analysis, prosthetics and clinical care, biomechanics, and consumer representatives that could, altogether, inform QALS management about the relevance and feasibility of research proposals.
- Creating a stakeholder matrix to organize controllers, promoters, providers, and advocates who can influence the provision of BAP (**Figure 8**). The immediate benefit of this exercise was to identify as exhaustively as possible all local, interstate, national, and international stakeholders. This task also required to clearly define the “power” and “interest” of a stakeholder corresponding to its capacity to influence allocation of resources and to provide prosthetic and medical care, respectively. These matrices were most helpful in engaging and managing communication with all stakeholders (e.g., seek funding, present at conferences).
- Profiling the case-mix involved in a study by presenting the distribution of consumers according to demographics (e.g., sex, age, height, weight, body mass index), amputation (e.g., time since first amputation and BAP, cause, level, number of amputations, length of residuum), and access to care (e.g., distance between residence to providers and QALS) characteristics. This information was essential to characterize potential confounders and their impact on the provision of BAP. For example, knowing the distance between a consumer's residence and the closest service provider is critical to determine how access to care across a wide state can affect the quality of care. This characterization became valuable when discussing outcomes and writing papers.

## FUTURE WORK

Future research will be undertaken in a global environment characterized by:

- Stronger evidence of efficacy and safety. Since this project, the body of peer-reviewed literature focusing on rehabilitation, prosthetic fitting, efficacy, and safety has grown noticeably.^3,5,35,65,82–99^ Several studies justified the prescriptions of MPK and ESAR components (e.g., Goldilocks zone loading).^62,100–102^ Health-related quality of life tend to be reported with a small range of surveys easing cross-comparisons between studies.^7,8,57–64^ However, there are still no straightforward standardized ways to report harms.^48–50^ Infections are graded using multiple nonstandardized systems.^59,65^ The risks are yet to be fully satisfactorily resolved.^5,65,85,87–89^ Little is known about long-term outcomes (e.g., influence of aging issues). Altogether, it is difficult to ascertain whether direct skeletal prosthetic attachment relying on percutaneous osseointegrated implants will overcome the “decline effect” as described by Harris (2016).^103^
- The emergence of global ecosystem. We are also witnessing the formation of a global ecosystem including a set of organizations and services integrating a value chain for the delivery of BAP through various commercial models (e.g., consumers and carers, providers of prosthetic solutions, administrators of healthcare organizations).^80^ The development of this ecosystem is stimulated by strong consumers' appeal for BAP, clearer and more diverse clinical pathways (e.g., indications, distal weight bearing system) and opening the market (e.g., approval from the American Food and Drug Administration).^17,100,104,105^ However, some funding bodies such as government organizations, private health care, work cover, and insurance are hesitant to fully support the provision of BAP requiring stronger evidence from registered clinical trials to test the possible decline effect.

Future studies could confirm whether the delivery of BAP changes the scope of practice (e.g., skills, risks) and business models (e.g., effects on incomes) for all allied health professionals (e.g., prosthetists, physiotherapists, occupational therapists). The outcomes of this preliminary CUAs could assist in building plausible scenarios when designing subsequent comprehensive CUAs relying on complex Bayesian or Markov state transition models (e.g., provision of osseointegration options compared to wheelchair, crutches, liners, and ischial containment and subischial sockets).^44,72,74–79^ More in-depth analyses can be performed from healthcare perspective (e.g., reimbursement standards) including surgical (e.g., one-off and on-going cost for primary surgical implantation, refashioning of residuum, reamputation, reimplantation), rehabilitation (e.g., physiotherapy), medical (e.g., pain killers, antibiotics), and prosthetic (e.g., socket fittings, interim and definitive prostheses) care costs More holistic CUAs could reveal the true costs of infections and subsequent surgical revisions.^2,98,99^

Future studies should also focus on societal perspective (e.g., the impact of BAP on employment, productivity, living assistance costs). Finally, future studies should also consider consumer perspective, often neglected but equally relevant (e.g., gap fees, out-of-pocket expenses, overseas travelling costs, prosthetic components, medication).

## CONCLUSION

Over the last 20 years, I witnessed genuine interest in osseointegration morphing into international momentum, leading to the emergence of a global ecosystem slowly paving the way toward recognition of direct skeletal prosthetic attachments. However, there is a long way ahead before evidence justifies the effective and global adoption of bionic solutions. Hopefully, this work will be a valuable contribution. Practical information and benchmark figures are provided. We estimated that 24% of the barriers to the project were specific to QALS, while 39% of the facilitators were transferable to other organizations. Above all, we shared a working approach to justify and organize the provision of prosthetic care for bone-anchored lower limb prostheses from a government perspective. Ultimately, we hope this work will inform promoters of prosthetic innovations committed to making bionic solutions widely accessible to a growing population of individuals suffering from limb loss worldwide.

## CALL TO ACTION

- Encourage authors of health economic evaluations to make their datasets publicly available (e.g., Data in Brief) to facilitate secondary observational studies as well as literature reviews and meta-analyses,
- Inspire decisionmakers responsible of provision of prosthetic care in Australia (e.g., National Disability Insurance Scheme) and other jurisdictions worldwide to continue this research work and consolidate evidence-based policies for delivery bone-anchored prostheses and bionic solutions,
- Motivate national and international stakeholders to establish reference groups working toward collegially agreed procedure (e.g., costs, process) to support reasonable standards of prosthetic care for individuals fitted with bone-anchored prostheses and bionic solutions.

## DECLARATION OF CONFLICTING INTERESTS

The authors are in the view that there is no competing interests conflicting with the content of this manuscript.

## SOURCES OF SUPPORT

This study was partially funded by the Queensland Artificial Limb Service, Medical Aids Subsidy Scheme, Metro South Health, and Queensland Government Minister of Health.

## AUTHOR SCIENTIFIC BIOGRAPHY

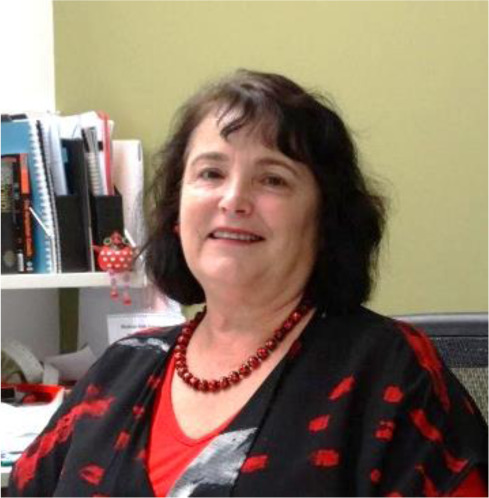



**Mrs Debra Berg** is the Manager of Queensland Artificial Limb Service, Queensland Health She has over 30 years' experience in Queensland services, including 20 years in delivery of artificial limbs. She is a strong advocate for bone-anchored prostheses in Australia for over a decade. Mrs Berg is acclaimed author of multiple reports and publications looking at the health service delivery and socioeconomics benefits of prosthetic osseointegration for individuals suffering from limb loss.

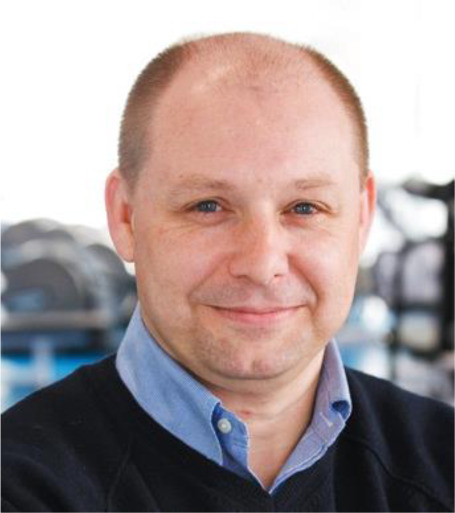



**Dr Laurent Frossard** is a bionic limbs scientist who is passionate about developing ground-breaking prosthetic solutions to improve the lives of individuals suffering from limb loss. He is internationally recognized as a researcher and an independent expert for his unique expertise in bionic limbs. He approaches bionic solutions from a holistic perspective, by integrating the prosthetic biomechanics, clinical benefits, service delivery, and health economics. Dr Frossard has over 25 years of experience, both in academia and in private industries in Australia, Canada, and Europe. He has collaborated with over 100 organizations worldwide. He is currently a Professor of bionics at the Griffith University, the Director and Chief Scientist Officer at YourResearchProject Pty Ltd, and Adjunct Professor at the Queensland University of Technology and the University of Sunshine Coast in Australia.

## LIST OF ABBREVIATIONS

BAP: Bone-anchored prostheses
CQI: continuous quality improvement
CUA: Cost-utility analysis
ESAR: Energy storing and return feet
HEE: Health economic evaluation
ICUR: Incremental cost-utility ratio
K: Medicare Functional Classification Level
MPK: Microprocessor-controlled knee
QALY: Quality-adjusted life-year
SF36: 36-Item Short Form Survey
SSP: Sockets-suspended prostheses
WTP: Willingness-to-pay threshold
